# Neonatal Resuscitation Skill-Training Using a New Neonatal Simulator, Facilitated by Local Motivators: Two-Year Prospective Observational Study of 9000 Trainings

**DOI:** 10.3390/children9020134

**Published:** 2022-01-20

**Authors:** May Sissel Vadla, Paschal Mdoe, Robert Moshiro, Ingunn Anda Haug, Øystein Gomo, Jan Terje Kvaløy, Bjørg Oftedal, Hege Ersdal

**Affiliations:** 1 Faculty of Health Sciences, University of Stavanger, 4021 Stavanger, Norway; bjorg.oftedal@uis.no (B.O.); hege.ersdal@safer.net (H.E.); 2 Haydom Lutheran Hospital, Haydom P.O. Box 9000, Mbulu, Tanzania; pfmdoe@gmail.com; 3 Muhimbili National Hospital, Dar es Salaam P.O. Box 65000, Tanzania; moshiror@gmail.com; 4 Laerdal Medical, 4002 Stavanger, Norway; ingunn.haug@laerdal.com (I.A.H.); oystein.gomo@laerdal.com (Ø.G.); 5 Department of Mathematics and Physics, University of Stavanger, 4036 Stavanger, Norway; jan.t.kvaloy@uis.no; 6 Department of Research, Stavanger University Hospital, 4011 Stavanger, Norway; 7 Department of Anaesthesia, Stavanger University Hospital, 4011 Stavanger, Norway

**Keywords:** neonatal resuscitation, simulation-based training, self-regulatory, Helping Babies Breathe second edition, feedback, implementation, motivators, training performance, ventilation quality, skill-training

## Abstract

Globally, intrapartum-related complications account for approximately 2 million perinatal deaths annually. Adequate skills in neonatal resuscitation are required to reduce perinatal mortality. NeoNatalie Live is a newborn simulator providing immediate feedback, originally designed to accomplish Helping Babies Breathe training in low-resource settings. The objectives of this study were to describe changes in staff participation, skill-training frequency, and simulated ventilation quality before and after the introduction of “local motivators” in a rural Tanzanian hospital with 4000–5000 deliveries annually. Midwives (*n* = 15–27) were encouraged to perform in situ low-dose high-frequency simulation skill-training using NeoNatalie Live from September 2016 through to August 2018. Frequency and quality of trainings were automatically recorded in the simulator. The number of skill-trainings increased from 688 (12 months) to 8451 (11 months) after the introduction of local motivators in October 2017. Staff participation increased from 43% to 74% of the midwives. The quality of training performance, measured as “well done” feedback, increased from 75% to 91%. We conclude that training frequency, participation, and performance increased after introduction of dedicated motivators. In addition, the immediate constructive feedback features of the simulator may have influenced motivation and training quality performance.

## 1. Introduction

Despite a global reduction in under-five child mortality over the past several decades, around 5.9 million children under five years die annually, with 2.7 million of these deaths occurring during the neonatal period [1]. Early neonatal mortality, i.e., deaths within the first seven days of life, constitutes 73% of neonatal deaths [2]. Intrapartum-related events, previously known as “birth asphyxia”, accounts for around 0.7 million of these cases, and approximately 1.3 million fresh stillbirths [3,4]. Many of these fresh stillbirths may be misclassified [3]. Therefore, in order to reduce perinatal mortality (i.e., fresh stillbirths and early neonatal deaths) it is crucial that health care providers possess adequate skills in neonatal resuscitation [5]. 

Simulation-based training in neonatal resuscitation is reported to be highly effective regardless of outcome variables, level of learner, and specific tasks being trained [6]. Another meta-analysis presents several features that are associated with increased effectiveness of high-fidelity simulators, such as provision of feedback, curriculum integration, range of difficulty level, repetitive practice, and individualized learning [7]. Barriers to simulation-based skill-training are typically high staff turnover and limited time to focus on training and practice [8]. However, it is also evident that a decline in knowledge and skills over time can be prevented by low-dose high-frequency refresher trainings, on-the-job practice, or similar interventions [8]. 

Helping Babies Breathe (HBB) is a simulation-based program for birth attendants in low-resource settings with the aim of improving skills in neonatal resuscitation. A low-cost NeoNatalie Newborn Simulator was developed (Laerdal Medical, Stavanger, Norway) to facilitate HBB skill-trainings [9]. The HBB First Edition was introduced in 2010 and is currently implemented in more than 80 low-income countries [10,11]. Several systematic reviews show convincing results regarding training performance, clinical practice, and reduced perinatal mortality following implementation of HBB [8,12,13,14]. However, midwives in rural Tanzania (i.e., Haydom Lutheran Hospital) have revealed that anxiety and fear of ventilating a non-breathing baby often led to poor resuscitation performance [15]. These midwives did not experience training with the original simulator, NeoNatalie, as sufficient to be optimally prepared for actual resuscitations, due to a lack of “sense of urgency” and missing responses from the simulator during training.

In 2016, the Second Edition of HBB was launched with an increased focus on quality improvement efforts and implementation strategies, including establishment of local facilitators to improve skills retention [10,11]. At the same time, a new, improved simulator, NeoNatalie Live (Laerdal Medical), was developed, featuring different patient cases, integrating HBB action points and scenario training, and automated responses from the simulator, including immediate feedback to the learners [16]. 

One recent multi-center observational study from Nepal has evaluated changes in practice following introduction of HBB Second Edition training and reported earlier initiation of neonatal ventilation after birth [17]. However, there are no studies evaluating the potential impact of the more advanced simulator, NeoNatalie Live, on training frequency and quality. Additionally, in previous studies, introduction of traditional HBB master trainers or instructors have been the case, but in line with the increased focus on quality improvement efforts in the Second Edition of HBB, we introduced a new role, “local HBB motivators”, to enhance the implementation process.

Implementation of the HBB Second Edition and NeoNatalie Live started in September 2016 at Haydom Lutheran Hospital. The objectives of the present study were to describe changes in staff participation in simulation skill-trainings, training frequency, and simulated ventilation quality over time, before and after the introduction of local motivators to facilitate HBB training from October 2017. 

## 2. Materials and Methods

This is a prospective observational study at Haydom Lutheran Hospital in rural Tanzania from 1 September 2016 to 31 August 2018. 

### 2.1. Study Setting

Haydom is a first referral hospital in rural Tanzania with 4000–5000 deliveries each year. The catchment area is approximately 2 million people. The hospital provides comprehensive emergency obstetric and basic emergency newborn care on a 24/7 basis [18]. Midwives largely conduct deliveries and neonatal resuscitation when indicated [19]. The labor ward holds six delivery beds and one operating theatre for caesarean sections. 

**Figure 1:**
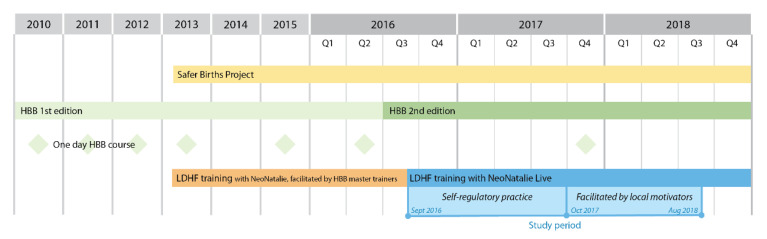
Simulation-based training at Haydom Lutheran Hospital 2010–2018. HBB; Helping Babies Breathe, LDHF; low-dose high frequency training. “Local motivators” were dedicated midwives tasked to facilitate and motivate for on-site LDHF training, following the HBB Second Edition.

HBB First Edition was introduced at Haydom in 2010. In 2013, the Safer Births project began, aiming to further improve HBB training and perinatal care [20]. In September 2016, the HBB Second Edition and NeoNatalie Live was introduced. All midwives were mandated to practice bag-mask ventilation on a weekly basis in addition to an annual one-day HBB course. An overview of simulation-based training at Haydom, prior to and throughout this study period, is presented in Figure 1. 

### 2.2. NeoNatalie Live—Newborn Resuscitation Simulator 

**Figure 2:**
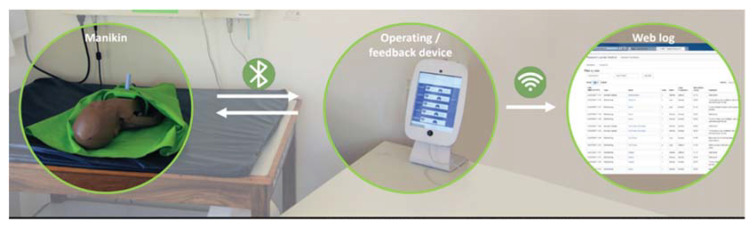
NeoNatalie Live including the manikin, operating/feedback tablet device (app), and web log. Photo by Laerdal Medical.

NeoNatalie Live is a substantially improved newborn resuscitation simulator, compared to the original NeoNatalie [16]. The manikin has a variable lung compliance, enables realistic bag-mask ventilation training, and has a dynamic heart rate that varies with lung aeration [21]. In total, four different patient cases, based on 1237 live resuscitations recorded at Haydom, can be trained [19]. The first patient case simulates a newborn with normal heart rate and normal lung compliance, while patient case four, the most complicated case, simulates low heart rate and stiffer lungs, representing water-filled or low compliant lungs. The simulator is operated by a tablet app where the learner registers and selects patient case (Figure 2). The training sessions last from 30 s to two minutes, depending on patient case and resuscitation/ventilation quality. After sufficient time with good quality ventilations, the mannequin starts crying, and the app provides automated feedback. The feedback suggests how to improve ventilation quality and encourages a new training attempt. The presented feedback is prioritized after the following list based on HBB; missing head tilt, insufficient opening ventilations, paused ventilations, mask leak, too high ventilation pressure, and ventilation rate. In sessions with several errors, only the most critical feedback is given. If the training scenario is repeated and the error is corrected, positive feedback is provided, followed by a new improvement advise. The feedback “well done” is provided when the learner conducts the training without any mistakes. The training session, including time and date, learner, and the full recording of the resuscitation is automatically up-loaded to and stored in a web log (Figure 2). “Valid ventilations” is defined as ventilations with correct head tilt and ventilation peak inflation pressure. This parameter does not include ventilation rate and pauses in ventilations. The percentage of “valid ventilations” is automatically calculated and presented in the web log. 

Both individual skill-training and scenario team training can be undertaken using the simulator. Only skill-trainings are included in this study.

### 2.3. Implementation of NeoNatalie Live and HBB Second Edition

Haydom implemented HBB Second Edition and NeoNatalie Live in their labor ward, September 2016, to enhance ongoing simulation training (Figure 1). Their main focuses were to reduce time to start ventilation and improve time of applied continuous ventilation and utilization of heart rate as an indicator of ventilation quality. During the first phase of the implementation, 1 September 2016–30 September 2017, the mannequin was used exclusively for individual skill-training according to self-regulatory practice. 

Initiating the second implementation phase, 1 October 2017–31 August 2018, the hospital management appointed four dedicated midwives to facilitate and motivate for training. These midwives, named “local motivators”, were responsible for encouraging midwives to train and for technical support to ensure an all-time functioning simulator. The local motivators were junior midwives, carefully selected according to their engagement in the ward. They were tasked to arrange scenario team-training and encourage the midwives to conduct self-regulated skill-training whenever time allowed. Initially, the motivators informed the midwives about potential benefits of simulation-based training in terms of increased confidence and improved skills in neonatal resuscitation. Thereafter, they reminded the midwives about training and answered questions on a daily basis. In addition, the group of midwives were divided in two teams, with two local motivators responsible for following up the training in each team. To increase motivation and training frequency, they arranged five informal competitions during the study period where participants on the winning team received a bottle of Coke. 

Regular skill-trainings were strongly recommended, but there were no specific rewards or negative consequences following the actual training participation or frequency for each midwife. The hospital management received monthly training-reports from the NeoNatalie Live data weblog, presenting training frequency and performance for the ward and the individual participants. All midwives registered on the NeoNatalie Live app using their true name, even though this was not mandatory. 

### 2.4. Study Participants

The study was conducted in a low-resourced clinical setting with a high turnover of midwives due to new government employment opportunities every midyear, leading to experienced midwives leaving the hospital and newly educated midwives starting at the end of each year. In total, 20 midwives participated from the beginning of the study, one midwife was enrolled in the study from 1 October 2017, and an additional nine midwives from 1 January 2018. During the study period, three midwives dropped out of the study from 1 January 2017, and two midwives were absent for a period of three months, 1 January 2017–31 March 2017. Consequently, the number of participants varied throughout the study period (September–December 2016; *n* = 20, January–April 2017; *n* = 15, May–September 2017; *n* = 17, October–December 2017; *n* = 18 and January–August 2018; *n* = 27). All midwives at the labor ward, and the neonatal unit from January 2018, were eligible for the study. All gave consent for participation in the study. This was not an obligation to participate in training. The participants were labeled “mandatory learners” in the period they were eligible (employed).

### 2.5. Data Collection

Data from all skill-training sessions were stored in the web log, providing detailed information about each training session registered between 1 September 2016–31 August 2018, except for December 2016, when the mannequin was out of order. The outcomes were participation of midwives, frequency of training, chosen patient case, percentage of trainings with feedback “well done”, valid ventilations, and ventilation rate. 

### 2.6. Statistics

Data were analyzed using SPSS version 26. Data were summarized by medians and quartiles. Non-parametric tests were used to compare the variables before and after appointment of motivators. A significance level of 0.05 was used in all hypothesis tests. Figures were produced in Excel.

## 3. Results

### 3.1. Participation and Frequency of Trainings Per Midwife

**Figure 3:**
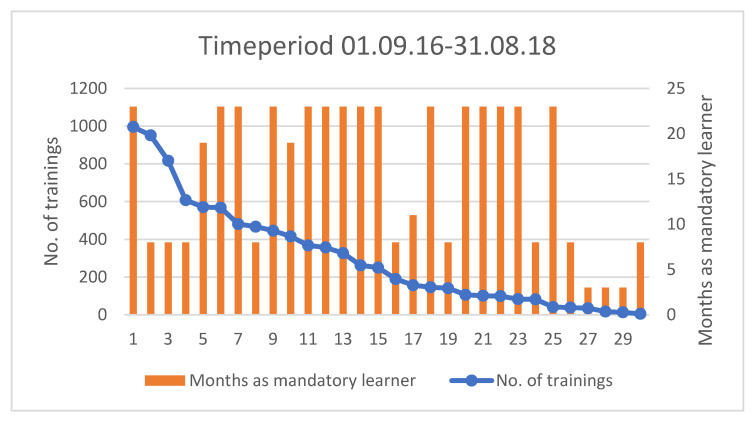
Descriptive data of training frequency for each midwife and duration of time classified as mandatory learner. The X-axis shows the individual midwives participating in the study, numbered from 1–30. The left Y-axis shows the number of trainings per midwife during their period as mandatory learner, illustrated by the blue frequency line. The midwifes are sorted according to this frequency.

In total, 30 midwives participated in this study. The number of trainings per included midwife through the study period and the time-period where they were classified as “mandatory learner”, varied among the midwives (Figure 3). For example, midwife number 3 completed 817 trainings and was employed at the labor ward for eight months during the study period.

The right Y-axis shows the number of months each midwife participated in the study, categorized as a mandatory learner, illustrated by the orange columns. 

### 3.2. Training-Frequency, Participation and Ventilation Quality

**Figure 4:**
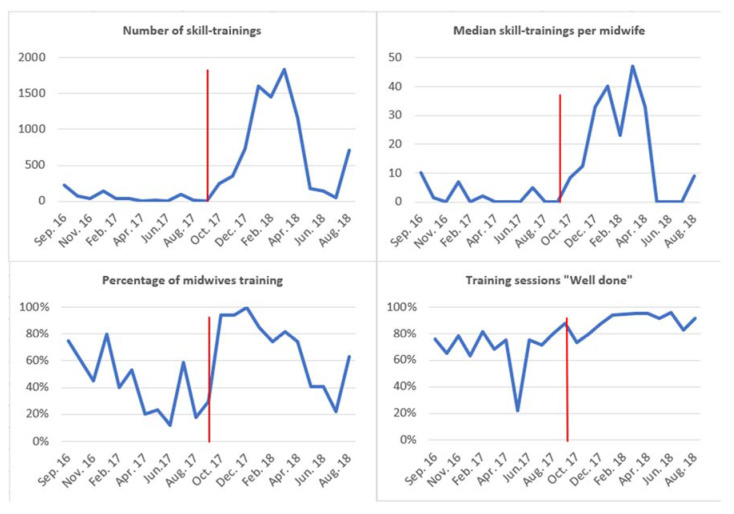
Timelines for number of skill-trainings, median number of skill-trainings per midwife, percentage of midwives participating in training and percentage of sessions receiving feedback “well done”.

Figure 4 describes the number of skill-trainings, median number of skill-trainings per midwife, percentage of midwives training, and percentages of training sessions receiving the feedback “well done” from September 2016 through August 2018. Local motivators were appointed in October 2017, illustrated by the red vertical line. 

The total number of skill-trainings increased from 688 during the 12 months with an operative mannequin before October 2017 to 8451 during the 11 months after the introduction of motivators in October 2017. There was a steep incline in number of skill-trainings from November 2017, with a marked decline in April 2018. The percentage of midwives conducting self-regulated trainings increased to almost 100% after introduction of local motivators, remained above 80% until April 2018, and decreased to 20% in July 2018. After November 2017, more than 90% of trainings were classified as “well-done”. All indicators appear to increase in August 2018 following a dip in July 2018.

Table 1 compares changes in number of mandatory learners (i.e., employed midwives), frequency of skill-trainings, participation of midwives (i.e., midwives undergoing training), performance of the trainings, and how often each patient case were selected, before and after appointment of motivators. 

The median number of mandatory learners and skill-trainings per month were substantially higher after October 2017 and appointment of local motivators. The number of monthly trainings per midwife increased from median 2.3 to 26.4 and the percentage of mandatory learners (midwives) undertaking training increased from median 43% to 74%. Training performance improved from median 75% to 91% classified as “well done”, and the percentage of valid ventilations increased from 98% to 100%. The results show no significant differences in ventilation rate and selection of patient cases between the two time periods. 

## 4. Discussion

This study indicates that it is possible to establish and maintain a culture of frequent in situ trainings in a hospital with limited resources, high turnover of midwives, and a low midwife-to-delivery ratio. The majority of midwives conducted self-regulated brief skill-trainings, using the NeoNatalie Live simulator, almost every day after appointment of local motivators. The number of simulation-based skill-trainings increased profoundly, and the percentage of newborn resuscitation simulations classified as “well-done” improved to and stabilized above 90%. 

No other relevant interventions or changes regarding neonatal resuscitation training was implemented at the hospital during the study period. 

### 4.1. Local Motivators and Training Frequency

The role of the local motivators in our study differs from more traditional master trainers or simulation facilitators who usually instruct in skill-training or facilitate team-simulations and debriefings. Our local motivators mainly motivated for training and provided technical maintenance of the mannequin. The latter is important in order to facilitate ongoing training [22], and preceding October 2017, the simulator was out of order for one month due to technical issues. This new “motivator” role has not been studied before in relation to newborn resuscitation training, as far as the authors are aware. 

Since 2011, Haydom has simulated low-dose high-frequency simulation-based HBB training to enhance newborn resuscitation care and specifically reduce challenges related to high staff turnover [18]. This study complements previous studies from Haydom demonstrating that high training frequency and participation (i.e., large proportion of midwives) are possible, even in a setting with low midwife-to-delivery ratio, high staff turnover, and limited resources [18,19]. These findings are contrary to previous literature stating all these aspects as barriers to training [8]. The present study indicates that the dedicated motivators managed to further increase training participation and frequency. Interestingly, the number of trainings and midwives conducting trainings declined markedly in the period with high turnover (April–July). The drop can also be a consequence of more focus towards scenario-based simulation training in the ward, conducted without the mannequin and thus not recorded in the data collection. All these circumstances may partly explain the decreased number of individual skill training between April–July 2018.

Other possible drivers for increased training, may be the strong management support and their acknowledgement of the motivators [22]. In addition, the midwives knew that the management could follow their participation and training frequency. Furthermore, it is likely that the long-lasting focus on HBB simulation-based training, placement of the simulator readily in the labor ward, and the short duration of the different patient cases (only a few minutes) contributed to the high training frequency, consistent with previous studies [23]. The improvement of resuscitation and ventilation skills following low-dose high frequency training is in line with other studies [18,19].

### 4.2. Features of the Simulator, NeoNatalie Live

Even though training frequency increased significantly after the introduction of motivators, still, 686 trainings were conducted during the first 12 months of the study. We speculate that the immediate constructive feedback from the simulator might have contributed to this high training frequency, consistent with other studies [7,24,25]. The automated feedback is related to the HBB action points, followed by recommendations on how to improve. However, it lacks some of the more comprehensive reflection elements that can be gained through facilitator-lead debriefing. A previous study showed that skill acquisition in cardiac compression training with automated feedback was not inferior when compared to a human instructor, and the majority of the participants found the automated mannequin feedback more useful than the instructor feedback [26]. The authors highlight that automated feedback in self-regulatory, individual skill-training, enables opportunities for learning with a potentially reduced need for human instructors. A review found anxiety related to being observed by peers during training perceived as a stressor in simulation training among nursery students, and automated feedback may facilitate a safer learning environment [27]. In addition to feedback, the realistic appearance of the mannequin and the different patient cases, may have contributed to enhanced motivation for training through increased relevance [7,21,28].

### 4.3. Optimal Level of Training Frequency

One important aspect regarding simulation-based training in neonatal resuscitation is the amount of training needed to improve and maintain skills and to translate acquired skills into clinical practice. A previous study indicates that an average skill drill of eight in three months leads to effective simulated ventilation, but more research is needed to find the optimal level of training frequency [29]. This is important for optimizing cost-benefit aspects of frequent on-site simulation-based training where time and resources are scarce, and training must fit in with clinical tasks. 

### 4.4. Strengths and Limitations

The strengths of this study are the long observation period of two years, the high number of trainings analyzed, and the automated and continuous measures of ventilation quality registered from every training session. 

This study is not a randomized controlled trial and cannot claim causality. Still, it seems likely that the increase in training frequency and participation among the midwives can be explained by the presence of dedicated motivators at the ward and the features of the simulator. Another limitation of the study is the new evaluation method of ventilation quality using data from the simulator in contrast to the frequently used OSCE score, making this indicator less comparable to previous studies. 

### 4.5. Future Studies

Skill-trainings with NeoNatalie Live aim to enhance the ability to follow the resuscitative HBB steps and mastery of bag-mask ventilations, which is fundamental to improve overall quality of newborn resuscitations [5]. Scenario team-trainings might be necessary to enhance clinical management, by increasing situation-awareness and decision-making regarding when to initiate resuscitation, and a rapid onset of ventilation when indicated. Further research should focus on using NeoNatalie Live for scenario-team training and investigate if the combination of individual skill-trainings and scenario-team trainings lead to improved clinical management of neonatal resuscitations and perinatal outcome. 

## 5. Conclusions 

Appointing dedicated motivators when implementing HBB Second Edition and NeoNatalie Live most likely increased training frequency, staff participation, and ventilation quality. 

Further research is needed regarding the underlying reasons for the increase in training, and whether this high training frequency had an impact on clinical management and perinatal outcome.

## Figures and Tables

**Table 1:** Changes in mandatory learners, training frequency, participation, training performance and selection of patient cases for simulation-based skill-training using NeoNatalie Live, comparing the period before and after appointment of local motivators.

	Before (1 September 2016–30 September 2017)	After (1 October 2017–31 August 2018)	*p*-Value
Mandatory learners, *n*	15–18	18–27	
Median per month (quartiles)	17.0 (15.0, 19.3)	27.0 (18.0, 27.0)	<0.001 ^†^
Skill-trainings, *n*	688	8451	<0.001 ^†^
Median per month (quartiles)	39.5 (8.3, 87.0)	713 (173, 1455)	
Skill-trainings/midwife/month, *n*			
Median (quartiles)	2.3 (0.5, 5.0)	26.4 (6.4, 53.9)	<0.001 ^†^
Midwives training, %			
Median (quartiles)	43 (21, 60)	74 (41, 94)	0.016 ^†^
Training performance Trainings “well done”, %			
Median (quartiles)	75 (66, 80)	92 (83, 95)	<0.001 ^†^
Valid ventilations, %			
Median (quartiles)	98 (87, 100)	100 (88, 100)	<0.001 ^†^
Ventilation rate, *n*			
Median (quartiles)	51 (45, 56)	51 (47, 54)	0.754 ^†^
Selected patient cases, *n* (%)			
Patient case 1	194 (28.2)	2154 (25.5)	0.131 ^‡^
Patient case 2	134 (19.5)	1924 (22.8)	
Patient case 3	147 (21.4)	1881 (22.3)	
Patient case 4	142 (20.7)	1760 (20.8)	
Random patient case	71 (10.3)	732 (8.7)	

^†^ Mann–Whitney U test, ^‡^ Chi-squared test.

## Data Availability

The data presented in this study are available on reasonable request to the corresponding author. However, we are not allowed to make these openly available due to regulations from the National Institute of Medical Research in Tanzania.
